# Composites of Rigid Polyurethane Foams Reinforced with POSS

**DOI:** 10.3390/polym11020336

**Published:** 2019-02-14

**Authors:** Sylwia Członka, Anna Strąkowska, Krzysztof Strzelec, Agnieszka Adamus-Włodarczyk, Agnė Kairytė, Saulius Vaitkus

**Affiliations:** 1 Institute of Polymer & Dye Technology, Lodz University of Technology, 90-924 Lodz, Poland; anna.strakowska@p.lodz.pl (A.S.); krzysztof.strzelec@p.lodz.pl (K.S.); 2 Institute of Applied Radiation Chemistry, Faculty of Chemistry, Lodz University of Technology, 90-924 Lodz, Poland; agnieszka.adamus@p.lodz.pl; 3 Vilnius Gediminas Technical University, Faculty of Civil Engineering, Institute of Building Materials, Laboratory of Thermal Insulating Materials and Acoustics, Linkmenu st. 28, LT-08217 Vilnius, Lithuania; agne.kairyte@vgtu.lt (A.K.); saulius.vaitkus@vgtu.lt (S.V.)

**Keywords:** POSS, ridgid polyurethane foams, mechanical properties, cellular structure

## Abstract

Rigid polyurethane foams (RPUFs) were successfully modified with different weight ratios (0.5 wt%, 1.5 wt% and 5 wt%) of APIB-POSS and AEAPIB-POSS. The resulting foams were evaluated by their processing parameters, morphology (Scanning Electron Microscopy analysis, SEM), mechanical properties (compressive test, three-point bending test and impact strength), viscoelastic behavior (Dynamic Mechanical Analysis, DMA), thermal properties (Thermogravimetric Analysis, TGA, and thermal conductivity) and application properties (contact angle, water absorption and dimensional analysis). The results showed that the morphology of modified foams is significantly affected by the type of the filler and filler content, which resulted in inhomogeneous, irregular, large cell shapes and further affected the physical and mechanical properties of resulting materials. RPUFs modified with APIB-POSS represent better mechanical and thermal properties compared to the RPUFs modified with AEAPIB-POSS. The results showed that the best results were obtained for RPUFs modified with 0.5 wt% of APIB-POSS. For example, in comparison with unfilled foam, compositions modified with 0.5 wt% of APIB-POSS provide greater compression strength, better flexural strength and lower water absorption.

## 1. Introduction

Rigid polyurethane foams (RPUFs) are highly cross-linked, three dimensional polymers with closed-cell structures which account for about 23% of all polyurethane (PU) production [1]. Due to their exceptional thermal-insulating properties, high resistance to weather conditions, good mechanical properties, and low apparent density [1], they are commonly used in industries such as furniture, automotive construction or in the production of thermal insulation materials [1,2,3,4]. RPUFs have become one of the most diverse and widely-used plastics with a continuously increasing global market. The total value of the global RPUF market amounts to 401 billion dollars and is expected to grow to 619 billion dollars in 2018 [5].

Beside the favorable properties of resulting materials, improving the mechanical properties of RPUFs is the foundation for their further potential applications. To successfully employ them in building application, it is critical that foams are combined with other materials or elements that provide mechanical strength and low thermal conductivity. A review of the related literature indicates that applying nanomaterials contributes a better improvement to mechanical properties and heat resistance [6,7,8,9,10,11,12,13,14,15,16]. Cao et al. [13] used an organically-modified montmorillonite to enhance several properties of RPUFs. The presence of nanoclay resulted in a reduction in cell size compared to a pristine RPUF sample. In the nanocomposites RPUF which used high molecular weight polyols, compressive strength dropped by 650%, but *T*_g_ increased by 6 °C. Widya and Macasko [7] incorporated montmorillonite-based organonanoclay into RPUFs at a clay loading of 1 wt%. They observed a reduction of mean cell size and a decrease in the diffusion of the blowing agent. Dolomanova et al. [11] investigated PU foam reinforced by single-walled carbon nanotube (SWNT) and multiwalled carbon nanotube (MWNT). The observation showed that both nanotubes improved compressive strength and compressive modulus, but, compared to SWNTs, MWNTs had a more remarkable improvement in the morphology and mechanical properties of the nanocomposite RPUF. This was because the MWNTs could interact with the matrix material and be better dispersed, which in turn affects the nucleation process and leads to a finer cell structure.

Alongside traditional substances, an interesting group of reactive nanofillers are polyhedral oligomeric silsesquioxanes (POSSs). POSSs are unique organic–inorganic nanobuilding blocks, with a rigid siliceous core with silicon (Si) atoms on the vertices and oxygen (O) atoms on the edges [17,18]. Organic groups are attached on the Si atoms, which allows the cage-like cores to be directly bound to polymer chains, via covalent linkage [19]. The cage POSS structures can be easily functionalized with various organic substituents, thanks to which they can participate in polymerization or grafting processes [20,21,22]. They show a versatile chemical compatibility owing to the diversity of organic substituents [23,24]. Due to the main features, such as hybrid nature and nanometer-sized structures with high surface area and controlled porosity, POSS compounds can be extremely attractive materials to be used as polyurethanes modifier [25]. Studies have shown that the incorporation of POSS molecules into various polymers can influence their thermal stability and degradation behaviour at elevated temperatures, change the glass transition temperature, increase the resistance to composite oxidation, and improve mechanical strength [22,25,26,27]. Among different POSS-containing polymer materials, hybrid PU-POSS elastomers of various architectures have been widely studied in the literature [17,18,19,28,29,30,31], but PU foam chemically reinforced by POSS constitutes a new class of materials.

The effects of open-cage nanostructure disilanoisobutyl POSS (DSI-POSS) on the properties of rigid PU foams were studied by Hebda et al. [32]. The hybrid composite foams containing 1.5 and 2.0 wt% DSI-POSS showed a reduced number of cells and an increased average area of foam cells in comparison with the unmodified PU. Thermogravimetric analysis results have shown that incorporation of POSS nanoparticles into PU foam does not significantly change the degradation process, however the POSS-modified samples, showed greater mechanical properties. Polyurethane foams reinforced by 1,2-propanediolizobutyl POSS (PHI-POSS) in the amount of 0.25, 0.5 and 0.75 wt% have been synthesized and investigated by Michałowski et al. [33]. The obtained results have shown that the use of PHI-POSS with phosphorus additive flame retardants leads to the reduction of the rigid polyurethane foam’s flammability, without significant changes of the foams’ crucial mechanical and thermal conductivity properties. Analogue tendency has been observed in the case of polyurethane foams modified with octa(3-hydroxy-3-methylbutyldimethylsiloxy) POSS (OCTA-POSS) [34].

The aim of this research is to develop and test PU foams modified with closed-cage nanostructure aminopropyl isobutyl-POSS (APIB-POSS) and aminoethylaminopropylisobutyl-POSS (AEAPIB-POSS). The influence of different amounts of POSSs on thermal properties (Thermogravimetric Analysis, TGA), dynamic mechanical properties (Dynamic Mechanical Analysis, DMA), physico-mechanical properties (compression strength, three-point bending test, impact strength, apparent density, and water absorption), and morphology of obtained PU composites was examined in the current work. The results obtained in the present work indicate that the addition of APIB-POSS and AEAPIB-POSS in the range of 0.5–5 wt% influences the morphology of analyzed foams and consequently their further mechanical and thermal properties.

## 2. Experimental

### 2.1. Materials and Manufacturing

**Figure 1:**
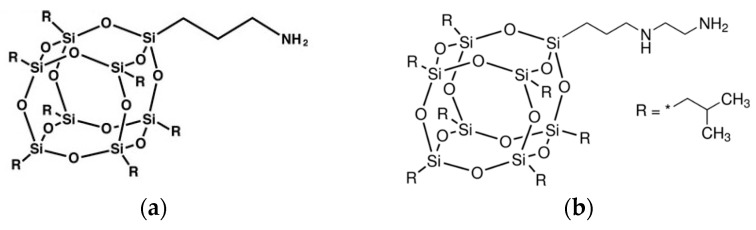
Chemical structure of (**a**) APIB-POSS and (**b**) AEAPIB-POSS.

**Figure 2:**
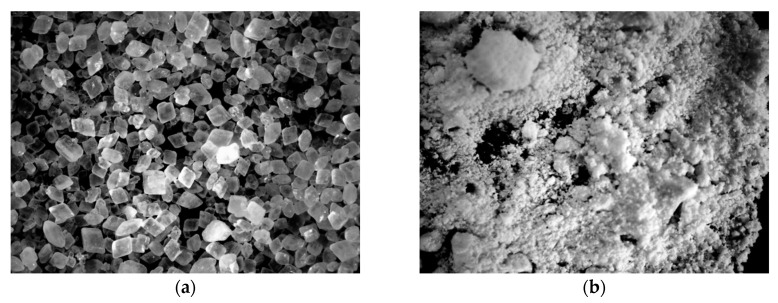
APIB-POSS and AEAPIB-POSS observed by a magnification of 50.

RPUFs were synthesized from the same polyurethane-modified polyisocyanurate rigid foam formulation based on petrochemical components provided by Purinova Sp. z o.o. at Bydgoszcz, Poland (Izopianol 30/10/C and Purocyn B). Izopianol 30/10/C used in the reaction is a fully formulated mixture containing a mixture of polyester polyol (hydroxyl number ca. 230−250 mg KOH/g, functionality of 2), catalyst (*N*,*N*-Dimethylcyclohexylamine), flame retardant (Tris(2-chloro-1-methylethyl)phosphate), and chain extender (1,2-propanediol) [29]. Purocyn B used in the synthesis is a polymeric diphenylmethane 4,4’-diisocyanate (pMDI) [29]. Aminopropylisobutyl-POSS (APIB-POSS) and aminoethylaminopropylisobutyl-POSS (AEAPIB-POSS) were provided by Hybrid Plastics Inc., Fountain Valley, CA, USA. The chemical structures of APIB-POSS and AEAPIB-POSS are presented in Figure 1a,b, respectively. Figure 2a,b shows the optical micrographs obtained for the APIB-POSS and AEAPIB-POSS, respectively. Since POSS molecules have polar groups and hydrophilic properties, strong interfacial interaction, such as hydrogen bonding, can be formed between the POSS molecules and isocyanate leading to the formation of a cross-linked structure. Amine groups present in POSS molecules can react with isocyanates even in the absence of catalyst [35,36,37,38]. It has been reported in previous work, that isocyanate has a higher reactivity with amines than with hydroxyl and carboxyl groups. The scheme of the amine reaction with isocyanate is shown in Equation (1). A urea bond is formed with this reaction. The generalized reaction scheme of isocyanate and POSS reaction is shown in Equation (1).

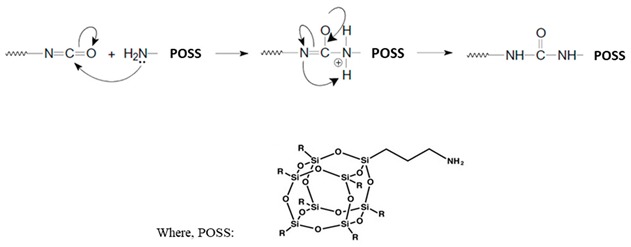
(1)

RPUFs were manufactured using a two-step method. In the first step, the component A, containing a polyol (Izopianol 30/10/C) and selected POSS, was prepared by mechanical stirring. The POSS reactive additive was introduced into the polyol in an amount of 0.5, 1.5 and 5 mass% of the polyol weight (wt%) and dispersed by the mechanical stirrer. In the second step, pMDI (Purocyn B) as component B was added to component A and the PU system was mixed using a mechanical stirrer for 10 s. After this time, the mixture was poured into an open mould where free foaming occurred in the vertical direction. RPUFs were conditioned at room temperature for 24 h. After this time, samples were cut with a band saw into appropriate shapes (determined by obligatory standards) and their physico-mechanical properties were investigated.

### 2.2. Characterization Techniques

The absolute viscosities of polyol and isocyanate were determined corresponding to ASTM D2930 (equivalent to ISO 2555) using a rotary Viscometer DVII+ (Brookfield, Germany). The torque of samples was measured as a range of shear rate from 0.5 to 100 s^−1^ in ambient temperature.

The apparent density of foams was determined accordingly to ASTM D1622 (equivalent to ISO 845). The densities of five specimens per sample were measured and averaged.

The morphology and cell size distribution of foams were examined from the cellular structure images of foam which were taken using JEOL JSM-5500 LV scanning electron microscopy (JEOL Ltd., Peabody, MA, USA). All microscopic observations were made in the high-vacuum mode and at the accelerating voltage of 10 kV. The samples were scanned in the free rising direction. The average pore diameters, walls thickness and pore size distribution were calculated using ImageJ software (Media Cybernetics Inc., Rockville, MD, USA).

The thermal properties of the synthesized composites were evaluated by TGA measurements performed using the STA 449 F1 Jupiter Analyzer (Netzsch Group, Selb, Germany). About 10 mg of the sample was placed in the TG pan and heated in argon atmosphere at a rate of 10 K min^−1^ up to 600 °C with the sample mass of about 10 mg. The initial decomposition temperatures, *T*_10%_, *T*_50%_ and *T*_80%_ of mass loss were determined.

The compressive strength (*σ*_10%_) of foams was determined accordingly to the ASTM D1621 (equivalent to ISO 844) using Zwick Z100 Testing Machine (Zwick/Roell Group, Ulm, Germany) with a load cell of 2 kN and a speed of 2 mm min^−1^. Samples of the specified sizes were cut with a band saw in the direction perpendicular to the foam growth direction. Then, the analyzed sample was placed between two plates and the compression strength was measured as a ratio of load causing 10% deformation of the sample cross-section in the parallel and perpendicular direction to the square surface. The result was the average of five measurements per each sample.

The impact test was conducted in accordance with ASTM D4812 on the pendulum 0.4 kg hammer impact velocity at 2.9 m s^−1^, with a sample dimension of 10 mm × 10 mm × 100 mm. All tests were conducted at room temperature, 25 °C. At least five samples were prepared for the tests.

A three-point bending test was carried out using Zwick Z100 Testing Machine (Zwick/Roell Group, Ulm, Germany) at room temperature, according to ASTM D7264 (equivalent to ISO 178). The tested samples were bent with a testing speed of 2 mm min^−1^. Obtained flexural stresses at break (*ε_f_*) results for each sample were expressed as a mean value. The average of five measurements per each type of composition was accepted.

Dynamic mechanical analysis (DMA) was determined using an ARES Rheometer (TA Instruments, New Castle, DE, USA). Torsion geometry was used with samples of that had a thickness of 2 mm. Measurements were examined in the temperature range 20−250 °C at a heating rate of 10 °C min^−1^, using a frequency of 1 Hz and applied deformation at 0.1%. 

Surface hydrophobicity was analyzed by contact angle measurements using the sessile-drop method with a manual contact angle goniometer with an optical system OS-45D (Oscar, Taichung City, Taiwan) to capture the profile of a pure liquid on a solid substrate. A water drop of 1 μL was deposited onto the surface using a micrometer syringe fitted with a stainless steel needle. The contact angles reported are the average of at least 10 tests on the same sample.

Water absorption of the RPUFs was measured according to ASTM D2842 (equivalent to ISO 2896). Samples were dried for 1 h at 80 °C and then weighed. The samples were immersed in distilled water to a depth of 1 cm for 24 h. Afterwards, the samples were removed from the water, held vertically for 10 s, the pendant drop was removed and then blotted between dry filter paper (Fisher Scientific, Waltham, MA, USA) at 10 s and weighed again. The average of five specimens was used.

Changes in the linear dimensions were looked into with accordance to the ASTM D2126 (equivalent to ISO 2796). The samples were conditioned at temperatures of 70 °C and −20 °C for 14 days. The change in linear dimensions was calculated in % from Equation (2).

Δ*l* = ((*l* − *l_o_*)/*l_o_*) × 100 (2)

where *l_o_* is the length of sample before thermostating and *l* is the length of sample after thermostating. The average of five measurements per each type of composition was reported.

## 3. Results and Discussion

### 3.1. Characterization of POSS-Modified Polyol Premixes

**Figure 3:**
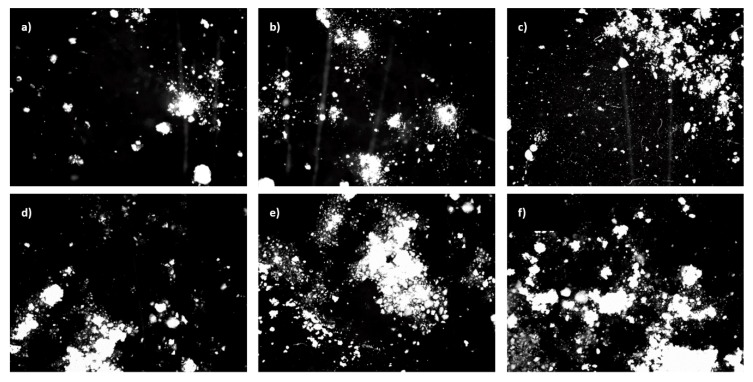
Polyol premixes with (**a**) 0.5 wt%, (**b**) 1.5 wt% and (**c**) 5 wt% of AEAPIB-POSS and (**d**) 0.5 wt%, (**e**) 1.5 wt% and (**f**) 5 wt% of APIB-POSS.

One of the most important parameters for industrial processing of polyols is their rheological behavior. The dispersion of the POSS’s particles in the PU matrix was evaluated by optical microscopy. Figure 3 shows the optical micrographs obtained for the samples with 0.5, 1.5 and 5 wt% of APIB-POSS and AEAPIB-POSS. A comparison of the optical images for 0.5 (Figure 3a) and 1.5 wt% of APIB-POSS (Figure 3b) with that of 5 wt% of APIB-POSS (Figure 3c) reveals that the higher concentration of particles is more difficult to disperse since the particles are closer to each other and will readily aggregate due to their high surface area and energy. An analogous trend is observed for premixes with AEAPIB-POSS (Figure 3c–e); however, the premixes show the accumulation of higher aspect ratio particles compared to the premixes with APIB-POSS.

The viscosity of the reactive mixture was measured first, since it is a critical parameter affecting the foaming process [39]. Increased viscosity hinders bubble growth, yielding foams with a lower cell size. Table 1 shows the viscosity of the polyol mixture plus the additives and fillers used in each formulation. The polyol premixes that contained APIB-POSS and AEAPIB-POSS are characterized by an increase in their viscosity with an increasing filler content, as a result of the presence of POSS particles interacting with the polyether polyol through hydrogen bonding and van der Waal’s interaction [40]. The effect of this higher-viscosity formulation is increased for RPUFs with AEAPIB-POSS.

**Figure 4:**
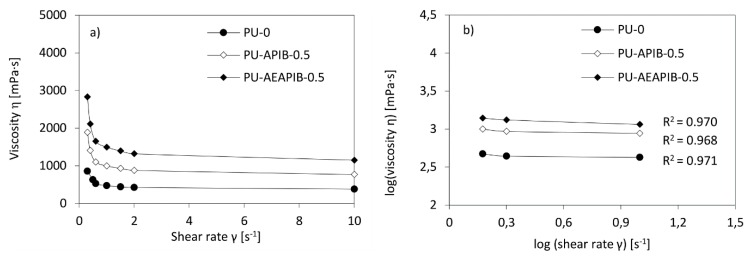
(**a**) Viscosity as a function of shear rate and (**b**) log-log plot of the viscosity vs. shear rate for the selected polyol premixes.

The rheological properties of polyol premixes are shown as the viscosity versus shear rate in Figure 4a. In all systems, the viscosity is generally reduced at increased shear rates. The viscosity of the samples initially decreases sharply and then significantly slows to reach a relatively stable value, due to the fact that particles of liquids reach the best possible arrangement. Such a phenomenon is typical for non-Newtonian fluids with a pseudoplastic nature and is found in many previous works [6,41]. To further analyze the data, the graph of viscosity versus shear rate is converted to log viscosity versus log shear rate form as shown in Fig 4b. From this graph, it can be seen that the curvatures of viscosity versus shear rate can be made close to linear using this log-log format with regression of 0.969–0.978. The power law index (*n*) was calculated from the slopes. All results are presented in Table 1. For the systems containing APIB-POSS, the power law index is lower than that of their AEAPIB-POSS-modified system counterparts. It indicates that the effect of the filler on the pseudoplasticity behavior becomes more significant for systems modified with APIB-POSS, leading to the highly non-Newtonian behavior.

The results presented in Table 2 indicate that with the incorporation of POSS into a polyol mixture, the maximum temperature of reaction (*T*_max_) slightly increases. It is believed that a higher temperature of the reaction of the modified compositions is connected with a higher reactivity of the components of the reaction mixture [42]. The presence of the additional groups as a result of the incorporation of POSS filler can lead to the exothermic reaction (see Equation (*1*)) providing more heat evaporation to the system, and consequently a higher temperature of the modified system compared with the PU-0 foam. It should also be pointed out that amine groups of the POSS can act as a catalyst and promote the polymerization reaction between polyol and isocyanate, which is an exothermic reaction [43,44,45]. This effect is most pronounced in the case of PU composites modified with 0.5 wt% of APIB-POSS and AEAPIB-POSS. With further increasing the concertation of POSS, the maximum temperature during the foaming process decreases. It has been shown that during the synthesis of the foams, the viscosity of the system increased with the addition of the POSS, and thus, a reduction in the efficiency of the reaction may have occured. Basically, analogue tendency has been observed by Kairytė et al. [46], who tested waste ash as a filler and flame retardant in PU foams. The authors have stated that the temperature decrease of PU composites can be attributed to the assumption that fillers absorb part of the heat generated during the foaming reaction.

### 3.2. Apparent Density of RPUFs

Apparent density is an important parameter that influences the properties and performances of RPUFs. The values of density of prepared foams are presented in Table 2. In general terms, the apparent density tends to increase when the APIB-POSS and AEAPIB-POSS are added. The reference foam is characterized by an apparent density of 38 kg m^−3^. The apparent density for RPUFs increases from 40 to 43 kg m^−3^ and from 40 to 44 kg m^−3^ with an increase of APIB-POSS and AEAPIB-POSS content of 0.5 and 5 wt%, respectively. This effect can be explained by the analysis of the role of filler particles on nucleation and cell growth. The POSS particles act as nucleation sites promoting the formation of bubbles, and this is an increasing trend with nanoparticles content, but, at the same time, the growth process of the resulting cells is hindered by an increase of the gelling reaction speed, which is revealed in the greater viscosity. This results in bubble collapse and higher density foams. Moreover, it should be pointed out that another factor affecting the density of RPUFs is higher density of POSS (*ca.* 1.2 g cm^−3^) compared to the PU foam matrix. This resulted in the increase of the apparent density of studied composites, wich is also in agreement with the results reported in the literature [47,48].

### 3.3. Foaming Kinetic

The foaming process was determined by measuring the characteristic processing times, such as cream, extension and tack-free time. The results presented in Table 2 indicate a slight increase in cream and extension time for the RPUFs containing APIB-POSS and AEAPIB-POSS fillers in each amount. This dependence is mostly related to the fact that well-dispersed filler in the reaction mixture acts as a nucleating agent in the nucleation process, leading to a greater bubble formation and prolonged cream time [49]. Moreover, further growth of the resulting cells seems to be hindered by the increase in viscosity of modified systems (see Table 1), leading to prolonged cream and extension times, which has also been noted by other researchers [50]. The polymerization kinetics are also affected by the presence of amine groups incorporated with the POSS molecules. Amine groups present in POSS molecules can react with isocyanates even in the absence of a catalyst [35,36,37,38]. It has been reported in previous work, that isocyanate has a higher reactivity with amines than with hydroxyl and carboxyl groups [35,36,37,38]. Because of this, most NCO groups are consumed in the formation of polyurea, and the number of NCO groups remaining to react with water is quite limited. This leads to less CO_2_ gases escaping from the foam structure and thus, to a prolonged start and gel time. Compositions modified with the addition of POSS are also characterized by a shorter tack-free time, indicating that POSS particles act as a curing accelerator. The total characteristic times measured for the POSS-modified compositions are higher than those measured for the PU-0, but still in the range of operation conditions for preparing RPUFs [51,52]. Contrary results were obtained by Liu et al. [17] who determined that compositions modified with waste ash were characterized by a longer tack-free time as well, indicating that the waste ash did not act as a curing accelerator. The authors have stated that this might be related to the fact that not all fillers determine the same reaction manner that is related to the chemical composition and particle size distribution leading to different foaming kinetics. 

### 3.4. Cell. Structure of RPUFs

**Figure 5:**
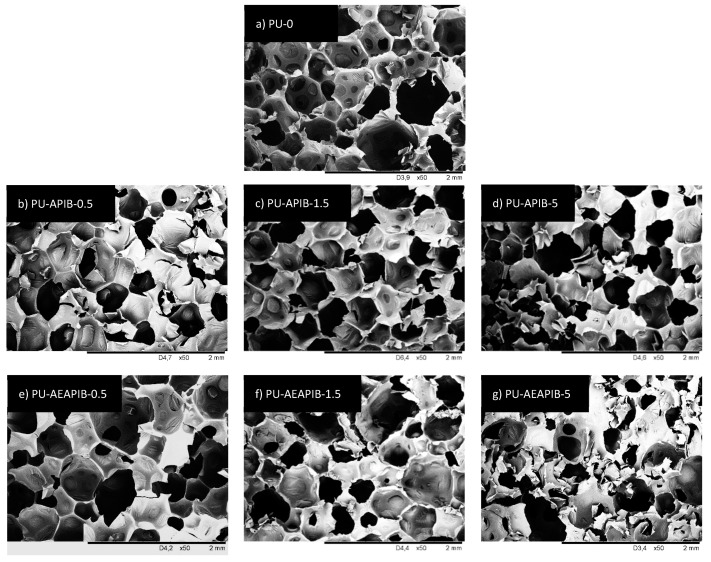
Morphology of (**a**) PU-0, (**b**) PU-APIB-0.5, (**c**) PU-APIB-1.5, (**d**) PU-APIB-5, (**e**) PU-AEAPIB-0.5, (**f**) PU-AEAPIB-1.5, and (**g**) PU-AEAPIB-5 observed at the same magnification.

The morphology of the RPUFs is shown in Figure 5, showing polygon closed-cell structures with many windows. The values of the cell sizes of the foams were statistically analyzed by means of *ImageJ* software from SEM images and the median values are summarized in Table 2. 

**Figure 6:**
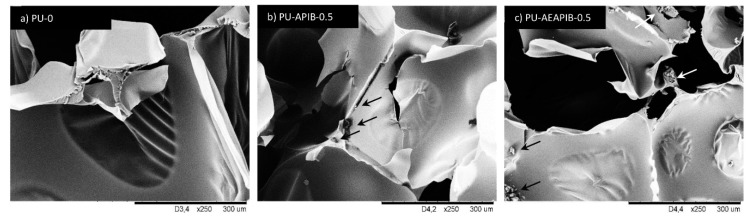
Morphology of (**a**) PU-0, (**b**) PU-APIB-0.5 and (**c**) PU-AEAPIB-0.5 observed at the same magnification.

As observed from the micrograph of the PU-0 (Figure 5a), the cell size and cell distribution are nearly uniform and the foam consists of closed cells with a negligible amount of cells with broken walls. Micrographs of the RPUFs with APIB-POSS are shown in Figure 5b–d. With the addition of APIB-POSS, the overall cell structure became less uniform and the amount of broken cells increased. The filled foams do not exhibit pronounced preferential orientation either; however, they are more irregular and a defective shape of cells with many cracks is observed. The morphology of PU-APIB-0.5 (Figure 5b) is mostly comparable to the PU-0, but with a further increase in the POSS content up to 1.5 wt% (Figure 5b), the overall structure becomes less uniform with a greater content of open cells and noticeable voids present in the structure. This trend is further prominent at 5 wt% filler content (Figure 5d), where an inhomogeneous structure and defective shape of cells with many cracks is observed. A similar trend is observed in RPUFs with AEAPIB-POSS, as shown in Figure 5e–g. With the increasing weight percent of AEAPIB-POSS, the cell distribution is transformed into non-uniform shapes and damaged cells appear. The explanation may be found in poor interfacial adhesion between the filler surface and polymer matrix, which promotes earlier cell collapsing phenomena and increases the high probability of generating open pores [53]. Moreover, the possible interphase interactions between POSS particles and polyurethane in cell struts disturbs the formation of a stable, non-defective foam structure [54]. The dispersion of the particles of used fillers in RPUFs is presented in Figure 6. It is clearly visible that for both series of modified foams, POSS particles are attached to the cell wall. Some dots and projections also become detectable in the cell void and a coarse surface can be seen in the cell struts.

**Figure 7:**
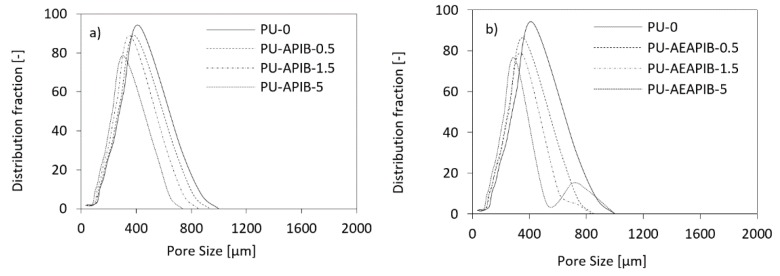
Cell size distributions of RPUFs modified with (**a**) APIB-POSS and (**b**) AEAPIB-POSS.

Table 2 presents the average cell sizes for the prepared PU foams. The addition of POSS into the PU matrix up to 5 wt% leads to a noticeable decrease in cell size, which can be also seen in the SEM images presented in Figure 5. It seems that filler particles can act as gas nucleation sites during the foaming process and assist in the formation of nucleation centers for the gaseous phase [53], thus affecting local rheology surrounding the growing bubbles [7]. The addition of powder filler can change the nucleation mode from homogenous to heterogeneous and reduce the nucleation energy, which in turn promotes the formation of large numbers of small cells [53], increasing the tendency of cell coalescence and leading to higher inhomogeneous cell size distribution. Comparing Figure 7a with Figure 7b, it is obvious that the incorporation of APIB-POSS resulted in smaller cells and a more homogenous structure as compared to their AEAPIB-POSS-modified RPUFs counterparts. In the case of RPUFs modified with APIB-POSS, most pores are located in the range of 300-350 µm. In the case of APIB-POSS-modified RPUFs, two populations of pores can be distinguished: large ones with a diameter of about 800 µm and small ones whose size is about 300 µm. A similar tendency has been reported in other studies [55,56,57].

### 3.5. Compressive Strength of RPUFs

**Figure 8:**
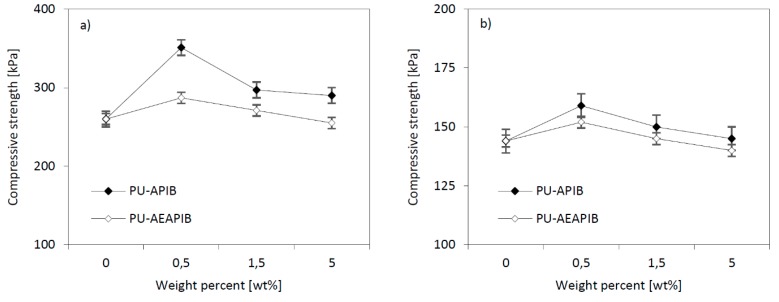
Effect of POSS content on the compressive strength of RPUFs measured (**a**) parallel and (**b**) perpendicular to the foam rise direction.

Another important parameter that impacts performance characteristics is the compressive strength, and the change in its value are presented in Figure 8a,b. The compressive strength of all materials tested in the direction parallel and perpendicular to the direction of foam rise is greater than the strength of the reference foam. In the case of foams with APIB-POSS, the largest increase in compressive strength is observed for the 0.5 wt% load (compared to the PU-0) and it is about 351 kPa in the parallel direction and 159 kPa in the perpendicular direction. In the foams containing 1.5 and 5 wt% of APIB-POSS, there is a decrease in compressive strength compared with RPUFs containing 0.5 wt% of APIB-POSS; however, it is still larger than for the reference foam. A similar trend is observed for foams modified with AEAPIB-POSS. With an increasing concentration of the modifier, the compressive strength decreases from 287 to 255 kPa in the perpendicular direction and from 152 to 140 kPa in the parallel direction. Nonetheless, similar to the APIB-POSS counterparts, the compressive strength of AEAPIB-modified foams is higher compared to the one of reference foams, except for the foam modified with 5 wt% of AEAPIB-POSS.

Such changes in the mechanical properties of composite samples can be explained in terms of characteristic features of their structure. As presented in Figure 5a, reference foam has a mostly spherical and equally distributed cell structure. With increased filler content, it could be observed that foam cell structure becomes more distorted and has a less uniform distribution. At this time, if there is an application of loading, bending and shrinkage of cell walls occurs and results in the development of micro cracks. Therefore, foam strength extremely depends on the initiation of micro cracks and forces on their growth [25]. So it can be explained by the decreasing foam compressive strength with crack initiation and growth.

Moreover, the reason for the decreasing mechanical strength at a high filling rate might also be related to the non-uniform dispersion of particles and polyol mixture. A high tendency to aggregate filler particles, noticeable in the structure, leads to a weakened interfacial adhesion between the filler and effective active surface. In consequence, RPUFs are characterized by a microphase separation of the structure, which leads to the failure of samples in an unexpected manner at random locations in the samples. The non-uniform concentration of the filler in some regions contributed to the embrittlement effect of polymer structures, inhibiting the enhancement of the mechanical properties of RPUFs. By increasing the content of the POSS, the negative effects of the filler such as disruption of the formation of hydrogen bonds and reaction stoichiometry, the probability of the agglomeration of nanoparticles due to the increase of viscosity and inappropriate distribution of nanoparticles is increased. Therefore, the particles interaction with PU macromolecules is decreased and the mechanical properties are weakened. The poor interfacial adhesion between some particles, especially the loose ones as discussed above, the polymer matrix and the uneven dispersion of the filler may lead to the above results, as proven by other authors [58,59,60,61].

### 3.6. Impact Strength of RPUFs

**Figure 9:**
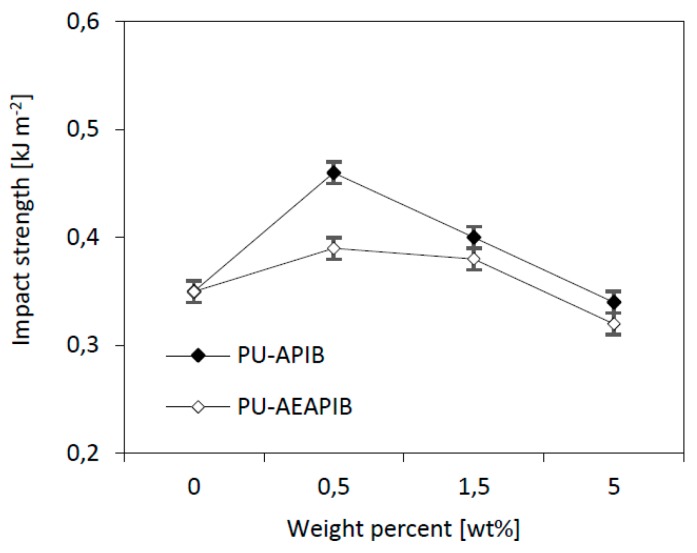
Effect of POSS’s content on impact strength of RPUFs.

With the increasing concentration of APIB-POSS and AEAPIB-POSS fillers from 0.5 to 5 wt%, the impact strength decreases from 0.46 to 0.31 kJ m^−2^ and from 0.39 to 0.32 kJ m^−2^ as shown in Figure 9. The impact strength shows a clear increase for RPUFs with APIB-POSS and AEAPIB-POSS by an amount of 0.5 wt% of the fillers. This behavior is related to the good interface reinforcement matrix and the generation of fracture paths through the POSS-reinforced RPUFs. The impact strength decreases with the increasing concentration of APIB-POSS and AEAPIB-POSS from 1.5 to 5 wt%. This result is caused by the POSS particles that serve as points for a localized stress concentration from which the failure begins or generally because of the elasticity reduction of the material with the POSS addition. Thus, the deformability of the RPUF’s matrix is reduced, which in turn affects the ductility in the foam surface. With this effect, the foam composite tends to form a weak structure and increase the concentration of POSS, thus reducing the foam’s energy absorption, resulting in reduced toughness and impact strength.

### 3.7. Flexural Strength of RPUFs

**Figure 10:**
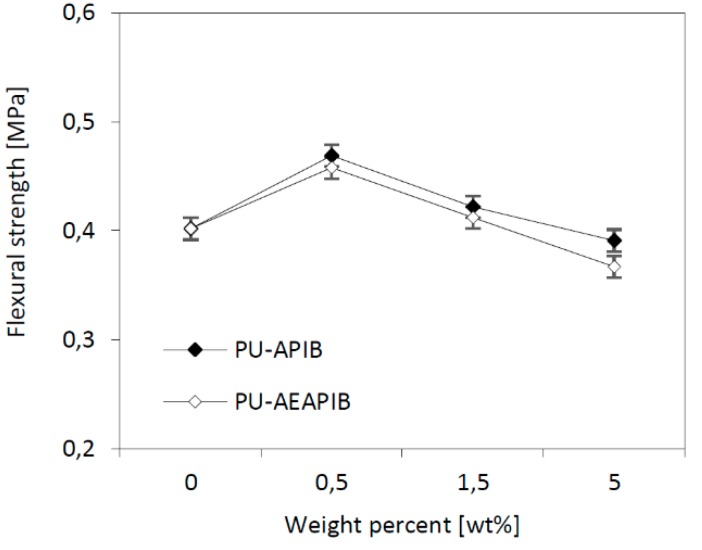
Effect of POSS’s content on flexural strength (*σ_f_*) of RPUFs.

The comparison between impact strength and tensile strength (*σ_f_*) shows that the same trend can be observed in both properties (Figure 10). Compared to the PU-0, RPUFs modified with 0.5 wt% of APIB-POSS and AEAPIB-POSS exhibits a slight improvement of the *σ_f_*. This behavior is attributed to the good interaction between the filler particles and PU matrix that delays the failure of the material until the mechanical strength of the fillers combined with the PU is reached. The incorporation of both fillers in the amount of 1.5 and 5 wt% leads to a deterioration of *σ_f_*, as a result of greater elasticity, connected with the cellular morphology of PU foams (see Figure 5). Due to an uneven distribution of the fillers in the matrix and many clusters present in the structure of modified materials, the mechanical properties of the resulting composite are reduced. The lack of a reinforcing effect with the incorporation of the filler was also observed in previous studies [62,63].

### 3.8. Dynamic Mechanical Analysis (DMA)

**Figure 11:**
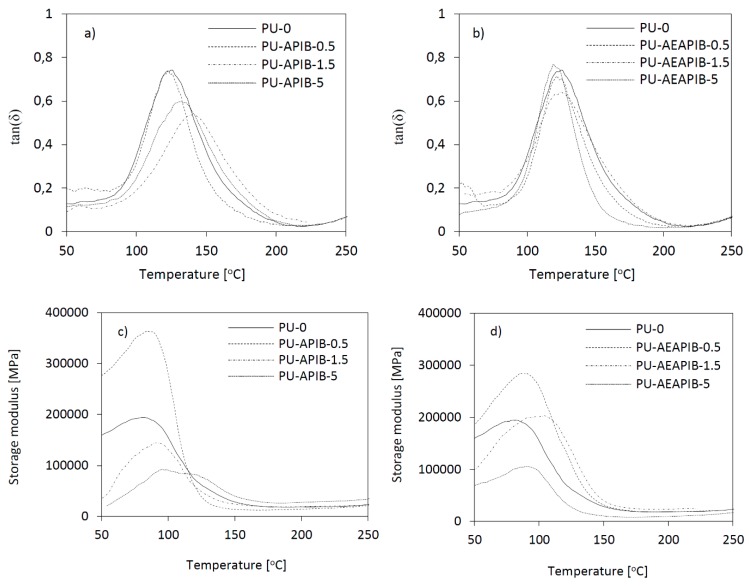
(**a**,**b**) tan*δ* and (**c**,**d**) storage modulus as a function of temperature plotted for RPUFs modified with APIB-POSS and AEAPIB-POSS.

RPUFs prepared with different weight percentages of APIB-POSS and AEAPIB-POSS were analyzed by DMA. The most interesting observation from this analysis is the displacement of the peak in the tan*δ* curves toward higher temperatures for samples modified with APIB-POSS and AEAPIB-POSS. The temperature of the maximum of the peaks associated to the main relaxation of the matrix is related to the PU glass transition temperature (*T*_g_). As shown in Figure 11a,b, the reference and POSS-modified foams exhibit one wide peak in the range of temperature analyzed. The width of the peak becomes broader with the POSS incorporation due to different relaxation mechanisms appearing in the modified materials as a consequence of the added filler. The broadening of the tan*δ* peak is often assumed to be due to a broader distribution in molecular weight between crosslinking points or heterogeneities in the network structures [17].

The comparison of PU-0 and POSS-modified composites shows that the addition of 0.5 wt% of APIB-POSS and AEAPIB-POSS results in an increase of storage modulus (*E′*), however, with the increasing content of the filler in the PU matrix, the value of the storage modulus significantly decreases (Figure 11c,d). This decrement in *E′* is attributed to the beginning of a thermal transition, which is associated with hard segments phase. The changes observed around 100 °C are attributable to the presence of a high concentration of hydrogen-bonded aromatic urethane groups in the poly(ether-urethane) phase and hard-segment domains which act as macroscopic cross links. The higher *E′* for RPUFs with 0.5 wt% of APIB-POSS and AEAPIB-POSS indicates more restricted mobility compared to the RPUFs with higher concentrations (1.5 and 5 wt%) of fillers; thus, the introduction of POSS over a certain optimal level, in this kind of RPUFs, produces more flexible materials. This might be connected with the fact that the PU matrix itself is highly crosslinked and an excess of POSS particles may actually disrupt this crosslinking, even if it is multifunctional. It has also been observed, that during the synthesis of the foams the viscosity of the system increased with the addition of the POSS (see Table 1), and thus a reduction in the efficiency of the reaction may have occured, leading to the more mobile polymeric network. It should also be pointed out that the higher viscosity of the system can also pronounce the aggregation of POSS at high loadings, and thus, the interaction with the matrix may be hindered.

### 3.9. Thermogravimetric Analysis (TGA)

**Figure 12:**
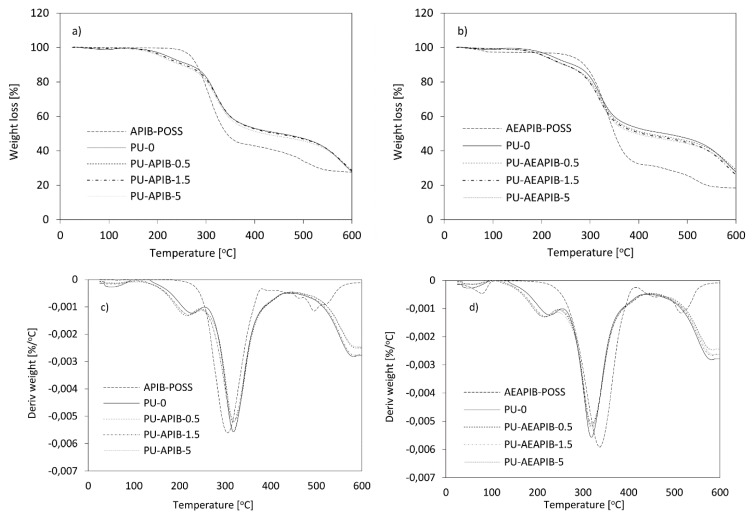
TGA curves for RPUFs modified with (**a**) APIB-POSS, (**b**) AEAPIB-POSS and DTG curves for RPUFs modified with (**c**) APIB-POSS and (**d**) AEAPIB-POSS.

The thermal stability of the RPUFs was characterized by thermogravimetric analysis (TGA) and derivative thermogravimetry (DTG), as illustrated in Figure 12a,b and Figure 12c,d, respectively.

Typically, the thermal degradation of RPUFs consists of three stages. The first step of decomposition is connected with the dissociation of the urethane bond at a temperature between 150 and 330 °C (corresponding to the temperature at 10% of total weight losses) [64,65]. The second step of the degradation of RPUFs occurs in the temperature range between 330 and 400 °C and is ascribed to the decomposition of soft polyol segments (corresponding to the temperature at 50% of total weight losses) [66]. The third step of degradation, ascribed to the degradation of the fragments generated during the second step, which corresponds to the loss of weight of about 80%, occurs at a temperature of 500 °C [64,67].

It has been noticed (Table 3) that there was no significant effect of POSS on the thermal stability of RPUFs, despite the fact that POSS compounds have a significantly different stability from PU-0. However, samples containing 0.5 wt% of POSS are characterized by the highest temperature at 70% weight loss, which indicates the POSS stabilizing properties. On the other hand, increasing the content of POSS causes more NCO groups to be involved in the formation of polyureas, and the number of NCO groups reacted with the polyol is quite limited. This leads to a reduction in the content of more thermally stable polyurethane groups compared to polyurea groups. The degradation of POSS-modified foams in the atmosphere of air runs in three distinct stages with maximum of mass loss at ca. 316–321 °C and 581–586 °C and involves the reaction of oxygen to form hydroperoxides which themselves are unstable and succumbing to decomposition to form more free radicals [68]. The slight increase in the char residues gives rise to the formation of more stable char layers, which may protect the materials from further decomposition and in turn increase the thermal stability. Compared to the foams modified with AEAPIB-POSS, the composites containing APIB-POSS are relatively less thermally stable. The degradation stage of the RPUFs modified with APIB-POSS starts at 245–247 °C, while the composite foams modified with AEAPIB-POSS start to degrade at slightly higher temperatures (248–251 °C). In both cases, for the highest filler content of 5 wt%, one may observe the deterioration of thermal properties. The decrease can be ascribed to the poor dispersion of the fillers and changes of the crosslinking density [6]. This is in agreement with the SEM images, where it is clearly visible that the presence of POSS increases the heterogeneity of RPUF’s morphology. The TGA results also suggest that the presence of POSS reduced the weight loss of RPUFs in the initial stage of degradation. This can be attributed to the barrier effect provided by POSS which reduces both the heat and oxygen fluxes toward the polymer surface, which limits the weight loss rate.

### 3.10. Contact Angle and Water Absorption

**Figure 13:**
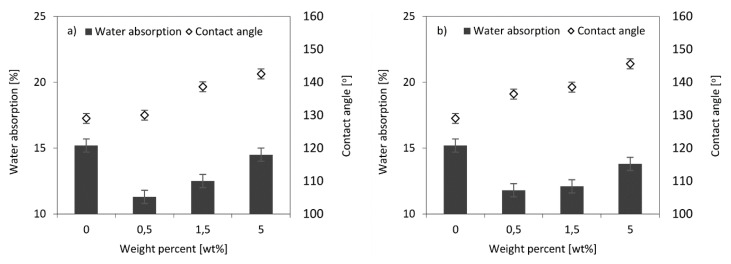
Effect of contact angle on water absorption of RPUFs modified with (**a**) APIB-POSS and (**b**) AEAPIB-POSS.

Polihedral oligomeric silsesquioxanes functionalized by amino groups significantly affected the hydrophobicity of the foams (Figure 13). Regarding water absorption, it is notable that foams modified by POSS absorb less water than the reference sample. This trend decreases with the increase in POSS content in the foam, but still, the affinity for water is lower for the POSS-modified RPUFs system. The use of both APIB-POSS and AEAPIB-POSS allowed the reduction of water absorption by almost one-third compared to the output foam without a modifier.

**Figure 14:**
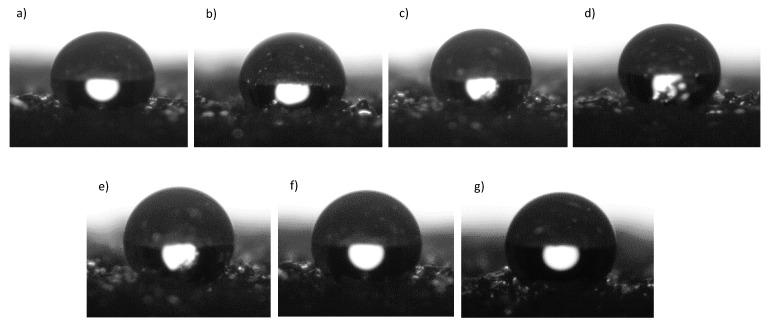
Contact angle of the surface of the (**a**) PU-0, (**b**) PU-APIB-0.5, (**c**) PU-APIB-1.5, (**d**) PU-APIB-5, (**e**) PU-AEAPIB-0.5, (**f**) PU-AEAPIB-1.5, and (**g**) PU-AEAPIB-5.

This tendency is explained by the hydrophobic character of isobutyl-POSS with reactive amino groups [69,70]. The contact angles (*θ*) of the RPUF composites with various weight percentages of APIB-POSS and AEAPIB-POSS also confirm this trend (Figure 14). It can be observed from the obtained results that by increasing both POSS contents, the water-contact angle of composite foams is increased. With a maximum of 5% of POSS, wettability was significantly reduced. This result implied the improvement of the foam hydrophobicity, leading to a limited absorption of water by the analyzed foams.

**Figure 15:**
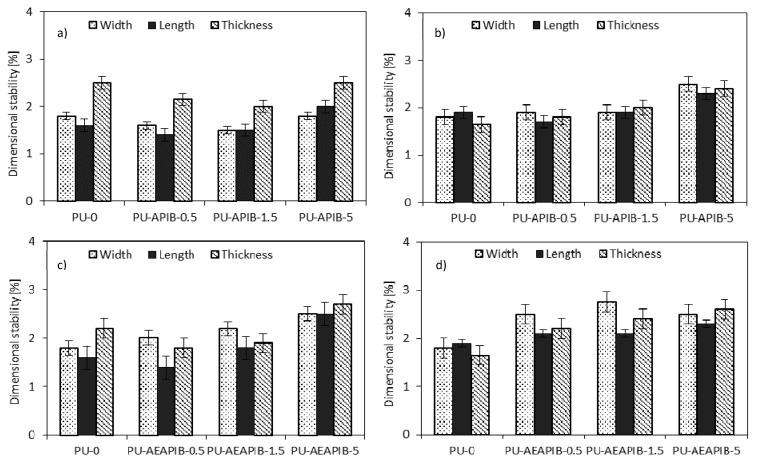
Dimensional stability of RPUFs modified with APIB-POSS (**a**,**b**) and AEAPIB-POSS (**c**,**d**) after exposure at 70 °C and −20 °C, respectively.

The % linear changes in length, width and thickness after exposure at 70 °C and −20 °C for up to 14 days for samples modified with APIB-POSS and AEAPIB-POSS are presented in Figure 15. The dimensional stability of RPUFs indicates that the addition of both fillers results in negligible changes of dimensional stability of the modified foams in relation to the PU-0. In all cases, the variations in the sample’s dimensions after the special treatment are random and thus, they can be attributed mostly to experimental errors while measuring. According to the industrial standard, the PU panels tested at 70 °C should have less than 3% of the linear change [71]. In each case, the dimensional stability of RPUFs is thus still considered to be mild and within commercially acceptable limits [71].

## 4. Conclusions

RPUFs were successfully reinforced using APIB-POSS and AEAPIB-POSS. The impact of POSS on the thermal properties, dynamic mechanical properties, physico-mechanical properties (compressive strength, three-point bending test, impact strength apparent density, and dimensional stability), foaming parameters, and morphology of RPUFs was examined. The presented results indicate that the addition of APIB-POSS and AEAPIB-POSS in the range of 0.5–5 wt% influences the morphology of analyzed foams and consequently, their mechanical and thermal properties. It was noticed that RPUFs modified with APIB-POSS are characterized by smaller and more regular polyurethane cells. This suggests better compatibility between the PU foam matrix and APIB-POSS, compared to the AEAPIB-POSS filler. This results in a significant improvement of the physico-mechanical properties and thermal stability of composites with APIB-POSS. For example, compared to the AEAPIB-POSS-modified RPUFs, composition with 0.5 wt% of the APIB-POSS showed greater compressive strength (351 kPa), higher flexural strength (0.458 MPa) and less water uptake (11% after 24 h). The results obtained in this study confirm that the addition of APIB-POSS and AEAPIB-POSS over a certain optimal level has a negative effect on cell morphology. The addition of both fillers in an amount of 5 wt% led to samples with reduced compression modulus, compressive strength, thermal transitions, and storage modulus with respect to the RPUFs containing 0.5 and 1.5 wt% of the fillers, mainly due to detrimental changes induced by the fillers.

## Figures and Tables

**Table 1:** Dynamic viscosity and logarithmic plot of the fitting equations for polyol premixes.

Sample Codes	Dynamic Viscosity *η* [mPa·s]			Fitting Equation	Power Law Index (*n*)	R^2^
	0.5 RPM	5 RPM	10 RPM			
PU-0	852	423	379	y = −0.058 + 0.335	0.335	0.970
PU-APIB-0.5	1887	878	765	y = −0.061 + 0.320	0.320	0.968
PU-APIB-1.5	3285	1542	821	y = −0.059 + 0.315	0.315	0.971
PU-APIB-5	6875	4250	1358	y = −0.059 + 0.295	0.295	0.972
PU-AEAPIB-0.5	2831	1317	1148	y = −0.058 + 0.325	0.325	0.979
PU-AEAPIB-1.5	4523	2015	1105	y = −0.060 + 0.315	0.315	0.971
PU-AEAPIB-5	7540	5250	1955	y = −0.062 + 0.310	0.310	0.976

**Table 2:** Selected properties of RPUFs.

Sample Codes	*T* [°C]	Cream Time [s]	Extension Time [s]	Tack-Free Time [s]	Cell Size [µm]	Wall Thickness [µm]	Apparent Density [kg m^−3^]
PU-0	110	43 ± 4	277 ± 10	341 ± 14	472 ± 10	62 ± 4	38 ± 1
PU-APIB-0.5	135	41 ± 2	312 ± 11	320 ± 12	390 ± 8	66 ± 2	40 ± 2
PU-APIB-1.5	128	46 ± 1	358 ± 12	265 ± 10	274 ± 7	68 ± 3	41 ± 1
PU-APIB-5	120	48 ± 2	370 ± 10	260 ± 12	209 ± 8	70 ± 2	43 ± 1
PU-AEAPIB-0.5	130	50 ± 2	332 ± 12	335 ± 15	364 ± 9	68 ± 5	42 ± 2
PU-AEAPIB-1.5	126	57 ± 4	360 ± 12	277 ± 14	320 ± 8	69 ± 4	44 ± 2
PU-AEAPIB-5	112	69 ± 3	384 ± 18	270 ± 16	184 ± 10	74 ± 4	40 ± 3

**Table 3:** The results of thermogravimetric analysis of RPUFs.

Sample Code	*T_g_*	*T_5_*	*T*_10_	*T*_50_	*T*_70_	Char Residue
	[°C]	[°C]	[°C]	[°C]	[°C]	[%]
APIB-POSS		267	280	342	531	21.6
AEAPIB-POSS		260	287	350	449	18.4
PU-0	126	220	265	454	591	27.9
PU-APIB-0.5	126	205	245	418	596	29.0
PU-APIB-1.5	129	206	247	406	590	28.6
PU-APIB-5	142	206	247	394	590	27.6
PU-AEAPIB-0.5	126	206	248	418	595	29.2
PU-AEAPIB-1.5	129	212	254	444	593	28.2
PU-AEAPIB-5	122	209	251	415	590	27.9
